# The influence of dark personality and pornography on sexual aggression beliefs

**DOI:** 10.3389/fpsyg.2024.1471438

**Published:** 2024-10-28

**Authors:** Manuel Galán, David Pineda, Pilar Rico-Bordera, Ana Martínez-Martínez, Jose A. Piqueras

**Affiliations:** Forensic Psychology Unit of the Centre of Applied Psychology, Department of Health Psychology, Miguel Hernández University of Elche, Elche, Spain

**Keywords:** dark tetrad, pornography, sexual violence, violence against women, latent profile analysis

## Abstract

**Introduction:**

Violence against women, particularly sexual violence, poses a significant public health concern. Predispositions toward perpetrating such acts often stem from the acceptance of myths that justify or deny these behaviours. This study aimed to explore how dark personality traits (narcissism, Machiavellianism, psychopathy, and sadism) and pornography consumption relate to the acceptance of these myths.

**Methods:**

Surveying 598 participants, the research employed Latent Profile Analyses (LPA) to identify distinct population profiles, Additionally, regression analyses were employed to further explore the relationships among variables.

**Results:**

Three profiles emerged, showcasing varying degrees of acceptance of sexual aggression myths. The most concerning profile, encompassing 9.2% of participants, displayed the highest alignment with these myths, alongside elevated scores in dark personality traits and pornography consumption. Notwithstanding the three profiles showed significant differences in the acceptance of these myths. Interestingly, the regression analysis highlighted that Machiavellianism stood out as the primary predictor for accepting sexual aggression myths, overshadowing the influence of pornography consumption.

**Conclusion:**

This emphasizes the role of personality traits in influencing attitudes towards sexual aggression myths. Moreover, implications for tailored prevention strategies, focusing on high-risk profiles, are discussed, highlighting the potential for targeted interventions to address harmful beliefs and behaviours.

## 1 Introduction

Violence against women, because of its implications, has been treated as a public health problem since 1996 (United Nations, 1996; World Health Organization, 2019). This violence can take different forms, including sexual violence. The World Health Organization (2013) defines sexual violence as being forced to have unwanted sex, to have sex out of fear of what the other person might do to you, and/or being forced to do something sexual that is humiliating or degrading to the victim. This situation can have significant physical, emotional, cognitive, and behavioural consequences for victims as for example, increased likelihood of PTSD, depression, anxiety, specific physical injuries, suicide, re-victimization, etc. (Classen et al., 2005; Barker et al., 2019). Understanding the factors that facilitate or predispose to this phenomenon is, therefore, necessary to prevent it.

### 1.1 Acceptance of modern sexual aggression myths

The acceptance of sexual aggression myths refers to generally false attitudes or beliefs that are widely accepted and used to deny or justify men’s aggressive behaviour against women (Lonsway and Fitzgerald, 1994). This set of attitudes or beliefs entails the denial of reality, minimization of the problem, and normalization of coercive attitudes (Gerger et al., 2007). All of this influences the normalization, facilitation, and execution of aggressive, hostile, and sexually violent behaviour towards women (Fernández-Fuertes et al., 2020; Samji and Vasquez, 2020; Trottier et al., 2021).

The effect of the acceptance of sexual aggression myths not only influences the perpetrators but also has effects on other actors involved in these situations, such as the victims, society, and the courts. In this case, victims may normalize aggressive situations or confuse what could be treated as rape for a sexual encounter based on their understanding of how a normative sexual encounter might take place (Ryan, 2011). Previous literature indicates that women who have experienced rape or sexual aggression are less accepting of these myths (Vonderhaar and Carmody, 2015). On the other hand, these myths affect courts and influence their decision-making, as for example, towards a ‘not guilty’ verdict, especially among individuals with stereotypical views about rape (Dinos et al., 2015; Leverick, 2020).

### 1.2 The role of pornography

Multiple social and cultural factors influence the acceptance of sexual aggression myths (Cooke et al., 2020; Trottier et al., 2021). Among other factors, it seems that new technologies can significantly influence how we see the world and relate to each other. Some studies have affirmed that the consumption of pornography might affect how we relate to each other sexually, reproducing the patterns we visualize (Wright et al., 2016; Rostad et al., 2019). Nevertheless, other authors argue that increased exposure to pornographic material does not affect attitudes towards sexual aggression on its own; it might influence in combination with different factors such as previous sexist attitudes, psychopathic or antisocial tendencies, low empathy, and alcohol use among others (Malamuth, 2018; Borgogna et al., 2022). Additionally, attempting to trace the mutual influences between the individuals and their environment from the confluence model, it has even been pointed out that these aggressive tendencies shape the preferred pornographic material and not the other way round (Seto et al., 2001). More specifically, a study by Borgogna et al. (2022) assesses the relationship between the use of generic and violent pornography and the acceptance of rape myths, which are less subtle than sexual aggression myths, and finds no clear and direct connection between these myths and pornography use.

### 1.3 The influence of personality

In this regard, as highlighted in previous paragraphs, sexual aggression is related to psychopathic or antisocial tendencies and low empathy among other factors. All of these factors are represented, to a greater or lesser extent, in the different traits that make up what has come to be known as the dark personality (Muris et al., 2017; Turner et al., 2019).

This dark personality construct refers to four personality traits, known as the Dark Tetrad personality (Paulhus and Williams, 2002; Chabrol et al., 2009). These four traits are subclinical narcissism, Machiavellianism, subclinical psychopathy, and everyday sadism. Subclinical narcissism defines a personality marked by feelings of grandiosity and importance, as well as a certain need for the approval of others (Raskin and Hall, 1981). Machiavellianism refers to a personality trait related to manipulation and the use of strategy; those with high scores on this trait tend to use others for their own benefit (Fehr et al., 1992). Subclinical psychopathy is related to people with impulsive tendencies, low empathy, and a lack of morality, among other characteristics (Hare, 1999). Finally, those with high scores in everyday sadism tend to enjoy the suffering of others, being the ones who inflict it or simply observe it (O’Meara et al., 2011).

These personality traits have already shown some relationship to pornography use (e.g., Burtăverde et al., 2021; Trapnell et al., 2024). More specifically, those scoring higher on the traits tend to view more pornography considered to be truly deviant (sex with minors and animals or rape) (Muris et al., 2020). On the other hand, dark personality has also been linked to a wide range of aggressive or violent behaviours, both sexual and non-sexual, including gender-independent outcomes (Pavlović et al., 2019; Pineda et al., 2021a). Additionally, different studies have shown that traits like psychopathy or Machiavellianism predict harmful attitudes toward intimate partner violence (IPV), as well as sexually violent behaviours. These traits, along with early adverse experiences, have been identified as significant contributors to violent and controlling behaviours in both general and incarcerated populations (Debowska et al., 2018; Waite and Mooney, 2024).

Furthermore, specifically addressing the relationship between these dark personality traits and the acceptance of sexual aggression myths has not been explicitly studied. However, to the knowledge of the authors, there are a few studies that have investigated the relationship between these traits and the acceptance of rape myths (e.g., Cooke et al., 2020; Lyons et al., 2022; Sánchez-Ruiz et al., 2021). For example, the study carried out by Sánchez-Ruiz et al. (2021) found that these traits predicted rape myth acceptance, although this relationship was moderated by gender beliefs. Explicitly, the authors found that the dark triad traits influenced masculine honour beliefs and hostile sexism, consequently influencing hostile attitudes. Longpré et al., 2022 also found a connection between the dark tetrad traits and rape myth acceptance, emphasizing the importance of the study of these links in order to develop effective intervention programs.

Further studies have examined the specific associations between each of these traits and constructs related to attitudes towards rape. For instance, the egocentric aspect of psychopathy, including deficits in the ability to mentally represent others’ cognitive responses and emotionally engage with them (cognitive responsiveness), has been found to predict positive attitudes toward rape or sexual aggression (Ioannides and Willmott, 2023; Willmott et al., 2024). Notwithstanding, in a recent review of all these connections, Costa et al. (2023) pointed out the necessity of exploring the relative effect of each of the tetrad constructs on this matter.

### 1.4 Person-centred analysis

Despite the growing body of research exploring personality traits and their influence on various behavioural outcomes, previous studies have predominantly relied on variable-centred analyses. These analyses focus on relationships between variables and often assume that patterns observed across groups apply equally to individuals who score highly or lowly on these traits (Wang et al., 2013; Von Eye and Wiedermann, 2015). However, this approach may overlook individual nuances or subgroup variations that could play a critical role in understanding complex phenomena like personality traits’ influence on behaviour.

Importantly, few studies have adopted a person-centred approach, which allows for the identification of subgroups of individuals who share similar patterns across multiple traits or behaviours (e.g., Rico-Bordera et al., 2024). Person-centred methods could offer more refined insights into how personality traits cluster within individuals and how these clusters are associated with outcomes, such as the acceptance of sexual aggression myths, yet these methods remain underutilized in the literature. Research in this area remains limited, with the majority of work focusing on variable-centred analyses and not fully exploring these individual-level patterns (Koehn et al., 2019).

Thus, our study employs both variable-centred and person-centred approaches to capture not only the broad trait-outcome relationships but also the nuanced variations within our sample. By combining these two methods, we aim to provide a more comprehensive understanding of how dark personality traits in combination with the behavioural variable of watching pornography influence the acceptance of sexual aggression myths. This approach addresses limitations in previous research that has not explored how these variables cluster or their varying effects on behaviour.

### 1.5 Objectives and hypotheses

With the aim of expanding our understanding of the interconnections between the introduced constructs, the present study endeavors to elucidate the relationships among dark personality traits, pornography consumption, and the acceptance of sexual aggression myths within the general population. For this purpose, from a person and a variable-centered approach, we pose the following research questions (RQs):

- RQ1: Are there different profiles based on pornography use and dark personality traits?
- RQ2: If there are different profiles, will there be differences among them in their acceptance of modern sexual aggression myths?
- RQ3: Are there gender differences in the study variables, and if so, will both genders be represented in all groups?
- RQ4: Which trait or traits will predict the most the acceptance of modern sexual aggression myths?

To answer the above questions, and based on previous literature, the following hypotheses (H) are put forward:

- *H1*: At least four different profiles are expected to exist depending on personality and pornography use: High dark personality traits and low pornography use; low traits and high pornography use; high traits and high pornography use; low traits and low pornography use; and average scores on all the variables.
- *H2*: Looking at the different profiles, it is expected that those with higher scores on both constructs (the dark traits and pornography use) will also have higher scores on the acceptance of sexual aggression myths than those with lower scores on pornography use or dark personality traits, and those with low scores on both.
- *H3*: Women will tend to score lower on all study variables (i.e., dark traits, pornography use, and sexual aggression myths acceptance) compared to men, as shown in previous research (Jones and Paulhus, 2014), but will also be represented in all the groups. Hence, while gender will impact the outcome, it will not stand as the sole predictive variable.
- *H4*: Machiavellianism and psychopathy will exhibit a stronger inclination towards accepting modern sexual aggression myths compared to the other dark tetrad traits.

## 2 Materials and methods

### 2.1 Participants

The survey initially reached a total of 1,537 participants; however, only 598 met the inclusion criterion of being over 18 and fulfilled the measures of interest, with 74% of these respondents being women. The sample collection used a convenience sampling method, utilising social networks such as Facebook, Instagram, and LinkedIn to access potential participants for the survey created with LimeSurvey.^1^ The age range of the participants was 18 to 80 years, with a mean age of 31.16 years (*SD* = 18.90). Regarding marital status, most were single at the time of the study (56%), 17.8% were married, 16.7% had a partner, and the remaining 10.5% were separated, divorced, widowed, or in other civil situations. In terms of educational level, a large proportion of the participants had completed a university degree or master’s degree (48.9%), followed by those who had completed a bachelor’s degree (36.1%); 8.9% of the participants had vocational training, 2.9% had a doctorate, 1.5% had completed primary education, 1.3% had completed secondary education, and the remaining 0.3% had no education.

### 2.2 Variables and instruments

The questionnaire filled in by the participants consisted of a first section with *ad hoc* socio-demographic questions such as age, gender, level of education, marital status, and so on. The following variables were then assessed.

#### 2.2.1 Dark personality

##### 2.2.1.1 Short dark triad (SD3)

The SD3 (Jones and Paulhus, 2014) scale was used to measure three dark personality traits—subclinical narcissism, Machiavellianism, and subclinical psychopathy. On this occasion, the Spanish version of the SD3 scale was used (Pineda et al., 2020). It is a 27-item instrument with a Likert-type response scale (0 = “Strongly Disagree” to 4 = “Strongly Agree”). Item examples are: “many group activities tend to be dull without me,” for narcissism; “it’s not wise to tell your secrets” for Machiavellianism; and “I avoid dangerous situations” for psychopathy. It consists of 9 items for each dimension or trait. For the current sample, the reliability values of the different scales were: α = 0.65, ω = 0.65 for narcissism; α = 0.77, ω = 0.77 for Machiavellianism; and α = 0.71, ω = 0.72 for psychopathy.

##### 2.2.1.2 Assessment of sadistic personality (ASP)

The Spanish version of the ASP (Plouffe et al., 2017) scale was used to assess the variable of everyday sadism (Pineda et al., 2021b). This instrument was designed to measure the construct of everyday sadism and can be used in combination with the previous instrument, SD3, to jointly assess the Dark Tetrad. In this case, it is an instrument with 9 items and a 5-point Likert-type scale (0 = “Strongly disagree” to 4 = “Strongly agree”). An example of an item is: “Being mean to others can be exciting.” The Spanish version of this instrument has already been used in combination with the SD3 in previous studies showing a multidimensional factor structure confirmed through Confirmatory Factor Analysis (CFA) and good psychometric properties (Pineda et al., 2021a, 2023). The reliability values for this scale in this sample were: α = 0.80, ω = 0.81.

#### 2.2.2 Use of pornography

Pornography use was assessed with a single item (“How often do you use pornographic material?”). Participants responded on a 5-point Likert-type scale (1 = Less than once a month, 2 = 1–2 times a month, 3 = 1–2 times a week, 4 = 3–4 times a week, 5 = 5 or more times a week). This item, and variations according to typologies, have been used in previous recent research (Rostad et al., 2019; Fisher and Kohut, 2020; Borgogna et al., 2022).

#### 2.2.3 Acceptance of sexual aggression myths

Acceptance of Modern Myths about Sexual Aggression scale (AMMSA; Gerger et al., 2007). The Spanish version of the AMMSA scale was used to measure the tendency to accept sexual aggression myths (Megías et al., 2011). It was developed to overcome the drawbacks marked by the social desirability of earlier scales such as the “Rape Myth Acceptance Scale” (RMAS; Burt, 1980). It is a 30-item scale with seven Likert-type response options (1 = “Strongly Disagree” to 7 = “Strongly Agree”). The Spanish version of the AMMSA (Megías et al., 2011) scale has adequate psychometric properties and a unidimensional structure α = 0.90. Our scale offered a five-factor factor structure similar to that initially theorized in the original study of the scale in English and German by Gerger et al. (2007). The factor analysis of the AMMSA in our sample derived the following dimensions: denial of the problem, consisting of 7 items from the original scale, an example is “Many women tend to exaggerate the problem of gender-based violence” and the reliability coefficients for our sample are α = 0.83, ω = 0.84; antagonism towards victims’ demands (antagonism), consisting of 4 items of the original scale, an example is “Instead of worrying about alleged victims of sexual violence, society should attend to more urgent problems, such as environmental destruction” and the reliability coefficients for our sample are α = 0.59, ω = 0.60; over-support, consisting of 3 items from the original scale, an example is “After rape, women today receive a lot of support” and the reliability coefficients for our sample are α = 0.70, ω =. 70; naturalization of coercion (coercion), consisting of 4 items from the original scale, an example is “When it comes to sexual contact, women expect men to take the initiative” and the reliability coefficients for our sample are α = 0.54, ω = 0.55; and, finally, exoneration of guilt (exoneration), consisting of 5 items from the original scale, an example is “For men, it is a biological necessity to release their accumulated sexual tension from time to time” and the reliability coefficients for our sample are α = 0.65, ω = 0.66. However, to allow for the comparison of our results with other studies in this area, calculations were also carried out using the one-dimensional proposal (Megías et al., 2011), obtaining reliability coefficients for our sample of α = 0.91, ω = 0.91.

### 2.3 Procedure

After the Ethics Committee approved the research, a cross sectional online survey was disseminated through the social networks stated in the participants section and messaging applications of the participating researchers. In turn, those who responded were also asked to share the survey. After giving their consent, participants responded to the instruments in the order previously presented. Participants did not receive direct compensation for completing the survey. However, they could request feedback on their personality profile and be entered into a prize draw for 10 Amazon cards worth 50 euros each. These measures were implemented to encourage the completion of the survey and enhance participant engagement. Additionally, the automated feedback and raffle procedures were ensured that the answers kept anonymous.

### 2.4 Data analysis

Initially, the reliability of the instruments (Cronbach’s alpha and McDonald’s omega) as well as the factor analysis of the AMMSA scale were conducted with the Jamovi (The Jamovi Project, 2021) following the recommendations of Kalkbrenner (2021). The same statistical package was used to obtain descriptive analyses, calculations of bivariate correlations, and comparisons between groups. Student’s and Fisher’s tests were used to analyze the differences between groups. Following (Cohen, 1988) suggestions, a η_p_^2^ equal to 0.009, 0.059, and 0.138, as well as a *d* equal to 0.2, 0.5, and 0.8 would be considered small, medium, and large effect sizes, respectively in both tests.

The latent profile analysis technique (LPA) was used to divide the sample into groups using MPlus (V. 8.6) (Muthén and Muthén, 2017). LPA is a technique that seeks to find profiles in the sample of people who respond in the same way to specified variables, in this case, the four traits of the Dark Tetrad and pornography consumption. To determine the most optimal clustering for the sample, fit indices were calculated for six possible models (from a single cluster to six different clusters). The fit indices used to find the solution were the Bayesian Information Criterion (BIC), the Akaike Information Criterion (AIC), the sample size adjusted BIC (SSA-BIC), the entropy, the Vuong-Lo-Mendel-Rubin index (VLMR), and the adjusted Likelihood Ratio Test (adjusted LRT). To determine the most optimal model, the significance of the VLMR, and adjusted LRT, a value close to 1 in entropy, as well as reduced AIC, BIC, and SSA-BIC values were considered (Spurk et al., 2020). Further, the Elbow graph was produced, which allows us to visualize the most optimal solution.

Furthermore, to further explore the results and account for other sociodemographic variables that could typically influence the outcomes, a regression model was constructed. The initial block encompassed sociodemographic factors (gender and age), while the second block incorporated both Dark traits and pornography use.

## 3 Results

With regard to the analysis of the variables (Table 1), direct correlations can be observed between all the variables studied. Participants’ gender shows the highest correlations with scores on aggression myth acceptance and pornography viewing, with males tending to score higher on these measures, confirming H_3_. Looking at the relationship between these two variables, acceptance of sexual aggression myths (total scale) and pornography use, an *r* of 0.21 is obtained (*p* < 0.01). In terms of the Tetrad traits, the trait most closely related to pornography use was psychopathy (*r* = 0.30, *p* < 0.01). The trait most closely related to the overall scale of acceptance of sexual aggression myths was Machiavellianism (*r* = 0.31, *p* < 0.01).

**Table 1:** Bivariate correlations and means and standard deviations of gender, pornography consumption, the dark tetrad traits, and acceptance of sexual aggression myths.

	*M (SD)*	(1)	(2)	(3)	(4)	(5)	(6)	(7)	(8)	(9)	(10)	(11)	(12)
Gender (1)		1											
Pornography consumption (2)	1.72 (1.06)	0.56^*^	1										
Narcissism (3)	13.61 (4.98)	0.13^*^	0.18^*^	1									
Machiavellianism (4)	16.04 (6.14)	0.17^*^	0.28^*^	0.33^*^	1								
Psychopathy (5)	8.53 (5.18)	0.27^*^	0.32^*^	0.40^*^	0.59^*^	1							
Sadism (6)	5.31 (5.06)	0.29^*^	0.30^*^	0.29^*^	0.59^*^	0.70^*^	1						
Denial of the problem (7)	17.66 (9.82)	0.28^*^	0.18^*^	0.19^*^	0.26^*^	0.25^*^	0.23^*^	1					
Antagonism (8)	12.00 (5.78)	0.20^*^	0.18^*^	0.20^*^	0.24^*^	0.23^*^	0.24^*^	0.70^*^	1				
Over-support (9)	8.60 (4.50)	0.27^*^	0.20^*^	0.13^*^	0.12^*^	0.13^*^	0.10^*^	0.52^*^	0.49^*^	1			
Coercion (10)	12.17 (5.26)	0.24^*^	0.14^*^	0.16^*^	0.26^*^	0.23^*^	0.21^*^	0.50^*^	0.44^*^	0.33^*^	1		
Exoneration (11)	13.11 (6.75)	0.18^*^	0.10^*^	0.18^*^	0.26^*^	0.23^*^	0.23^*^	0.63^*^	0.53^*^	0.39^*^	0.58^*^	1	
Acceptance of sexual aggression myths (12)	78.71 (30.68)	0.31^*^	0.21^*^	0.24^*^	0.31^*^	0.30^*^	0.28^*^	0.90^*^	0.81^*^	0.64^*^	0.69^*^	0.80^*^	1

For Gender: 1, female; 2, male.

^*^p < 0.01.

**Figure 1:**
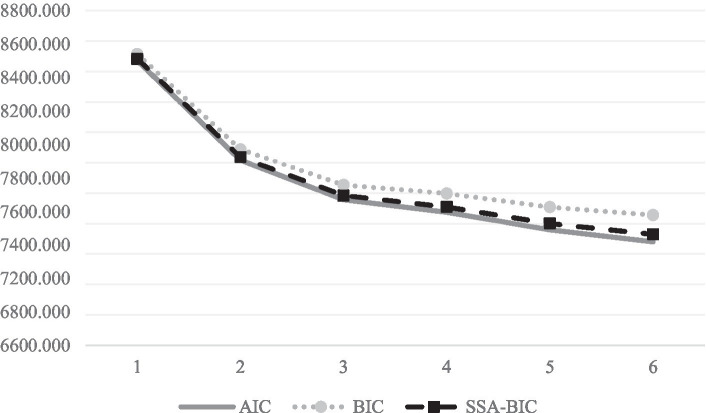
Elbow graph for the solutions from 1 to 6 profiles.

Regarding the latent profile analysis (LPA), Table 2 shows the fitting values for the solutions of 1 to 6 different profiles. After analyzing these results and the elbow graph’s results (Figure 1), the most optimal solution was the one with 3 different profiles or classes, partially fulfilling H_2_.

**Table 2:** Model fit indices for 1- through 6-profile solutions.

Profiles	AIC	BIC	SSA-BIC	Entropy	VLMR	Adjusted LRT
1	8470.703	8514.639	8482.892			
2	7816.712	7887.010	7836.214	0.787	0.0001	*p* < 0.001
**3**	**7556.933**	**7653.592**	**7583.749**	**0.854**	**0.0001**	*p* < 0.001
4	7474.554	7597.575	7508.683	0.881	0.1989	*p* > 0.05
5	7358.454	7507.836	7399.896	0.886	0.4217	*p* > 0.05
6	7279.831	7455.575	7328.586	0.904	. 1,204	*p* > 0.05

AIC, Akaike Information Criterion; BIC, Bayesian Information Criteria;SSA-BIC, BIC adjusted for sample size; VLRM, Vuong-Lo-Mendel-Rubin; LRT, Likelihood Ratio Test. Bolded values indicate the chosen solution.

**Figure 2:**
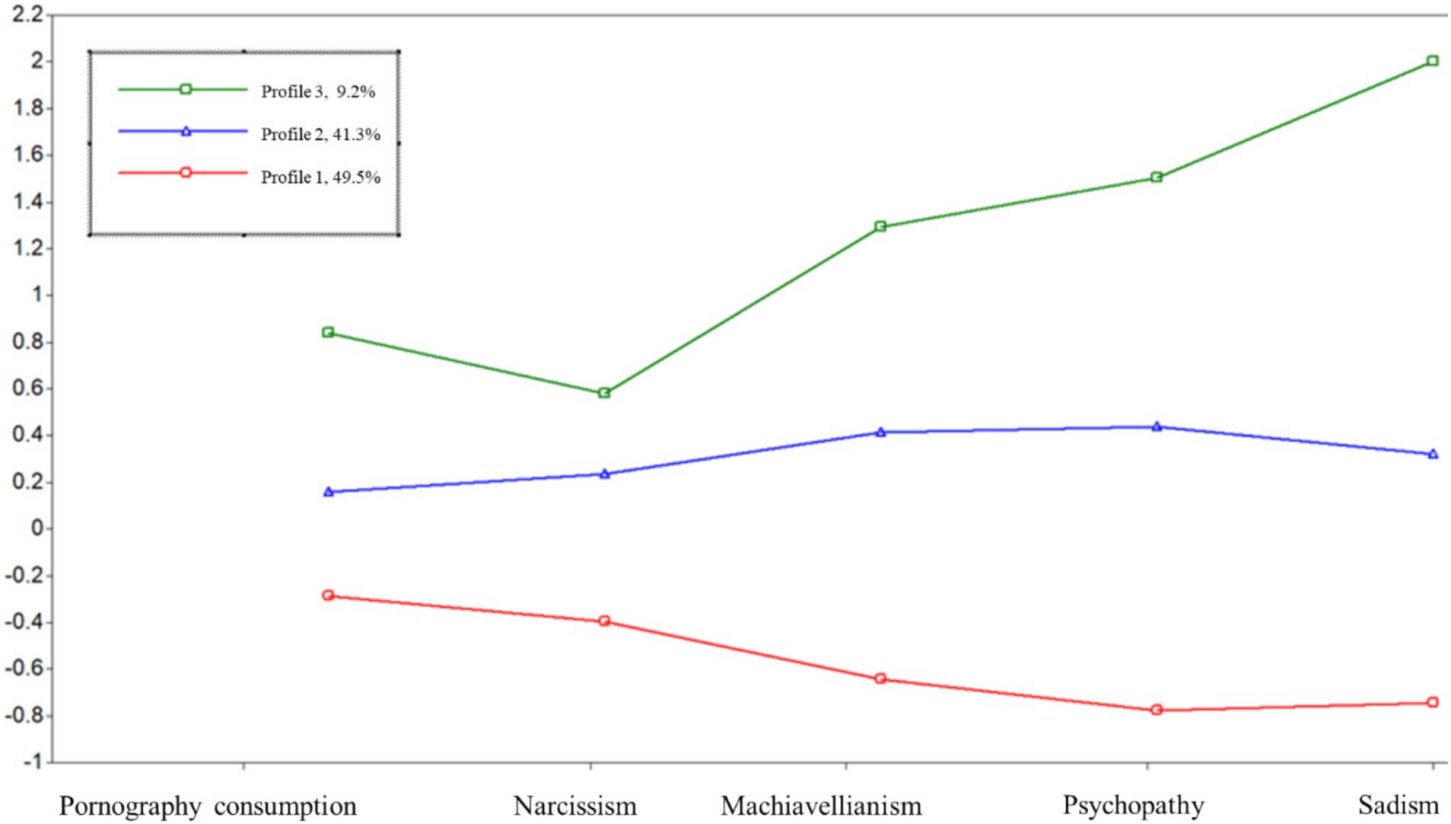
Mean values of pornography consumption and the dark tetrad traits as a function of the latent profile analysis.

Having obtained the most optimal solution for our sample, Figure 2 plots the standardized mean scores of the 3 profiles on the grouping variables (i.e., pornography consumption and Tetrad traits). It can be observed that the first profile, composed of most of the participants (49.5%), presents low scores on all variables, consuming little pornography and scoring low on all personality traits. On the other hand, the second group, made up of 41.3% of the sample, has scores very close to the average on all variables. Finally, the last group (profile 3) could be defined as the group with the most malice. This group has the highest scores in pornography consumption and in all the traits of the Dark Tetrad, with everyday sadism as the most marked trait.

The sample was divided into different profiles; Table 3 shows the existence of differences in the standardized scores between these subgroups. The mean effects of the differences between groups can be observed on the total scale (η_p_^2^ = 0.10), as well as in the denial dimensions of the problem (η_p_^2^ = 0.07), of antagonism towards the demands (η_p_^2^ = 0.08), and exoneration of guilt (η_p_^2^ = 0.06). *Post hoc* comparisons revealed significant differences among the three profiles, particularly evident between profiles 1 and 3 across all dimensions, and between 1 and 2 across all dimensions except for the over-support dimension. Groups 2 and 3 were more similar, only finding significant differences when considering the total AMMSA score and the dimension of antagonism towards demands.

**Table 3:** Differences in the standardised scores among the three profiles in the variables of interest.

Variables	*M (SD)*			*F*_(2,597)_	η_p_^2^	*p* Scheffe (Cohen’s *d*)		
	Profile 1 (*n* = 296)	Profile 2 (*n* = 249)	Profile 3 (*n* = 53)			1 vs. 2	1 vs. 3	2 vs. 3
AMMSA denial	−0.24 (0.86)	0.11 (1.00)	0.55 (1.04)	21.16^*^	0.07	<0.001 (0.39)	<0.001 (0.85)	0.009 (0.47)
AMMSA antagonism towards demands	−0.24 (0.89)	0.10 (0.96)	0.71 (1.14)	25.61^*^	0.08	<0.001 (0.36)	<0.001 (1.00)	<0.001 (0.64)
AMMSA over-support	−0.12 (0.95)	0.01 (1.02)	0.48 (0.99)	8.33^*^	0.03	0.344 (0.13)	<0.001 (0.61)	0.007 (0.48)
AMMSA coercion	−0.23 (0.88)	0.14 (0.99)	0.43 (1.13)	16.23^*^	0.05	<0.001 (0.38)	<0.001 (0.69)	0.123 (0.31)
AMMSA exoneration	−0.23 (0.86)	0.10 (1.01)	0.51 (1.13)	17.60^*^	0.06	<0.001 (0.35)	<0.001 (0.78)	0.019 (0.43)
AMMSA total	−0.27 (0.78)	0.12 (0.97)	0.72 (1.21)	33.11^*^	0.10	<0.001 (0.45)	<0.001 (1.11)	<0.001 (0.66)

*p < 0.01.

**Figure 3:**
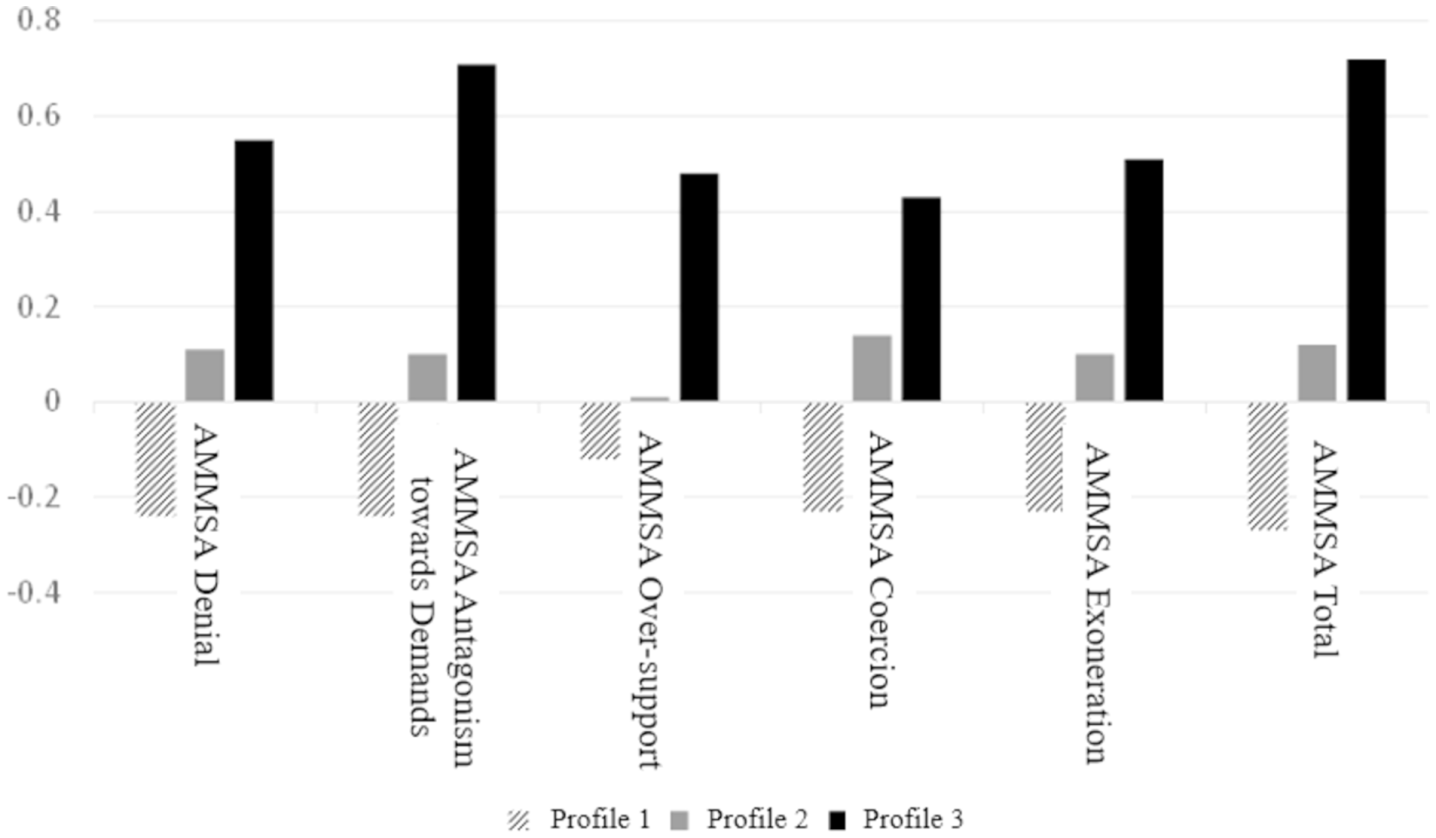
Mean scores of the profiles in the variables of interest.

These results, as can be seen graphically in Figure 3, show that profile 1 (participants who scored lowest on the Dark Tetrad and pornography use) have the lowest scores on all dimensions of the Acceptance of Sexual Aggression Myths scale, supporting H_2_. Conversely, profile 3 (those with the highest scores on pornography use and the Dark Tetrad) have the highest scores on the AMMSA scale, indicating greater acceptance of sexual aggression myths.

Gender differences, in line with H_3_, were observed in all variables included in the study (*p* < 0.01). The biggest difference, attending to the effect sizes, was observed in the use of pornography and the AMMSA total score variables (Cohen’s *d* = 1.56 and Cohen’s *d =* 0.73, respectively). Notwithstanding, women were present in the three groups of the LPA with 83.9% in Profile 1, 73.4% in Profile 2, and 35.2% in Profile 3, indicating an overrepresentation in groups one and two.

For a further analysis of these results, and aiming to test H_4_, a regression model was calculated with the total AMMSA score. The results are presented in Table 4. The trait that predicted AMMSA better was Machiavellianism, while sadism did not have an effect, as well as pornography use with no effect in this model. However, it is worth noting that gender also played an important role in this model accounting for a 3.61% in the observed variance change.

**Table 4:** The predictive capacity of the dark tetrad and pornography use for acceptance of sexual aggression myths (total score).

Predictor variables	Step 1				Step 2			
	β	*t*	*r_x.y_*	*sr* ^2^	β	*t*	*r_x.y_*	*sr* ^2^
Gender	0.30	7.65^*^	0.30	9%	0.22	4.71^*^	0.19	3.61%
Age	0.08	1.94	0.08	0.64%	0.12	3.20^*^	0.13	1.69%
Narcissism					0.11	2.74^*^	0.11	1.21%
Machiavellianism					0.17	3.53^*^	0.14	1,96%
Psychopathy					0.07	1.27	0.05	0.25%
Sadism					0.05	0.95	0.04	0.16%
Pornography use					−0.01	−0.97	0.00	0.00%
*R^2^_adjusted_*	0.10				0,18			
*F*	32.56^**^				20.11^***^			

*p < 0.01.

## 4 Discussion

The main aim of the present study was to clarify the relationships among the Dark Tetrad, pornography consumption, and the acceptance of sexual aggression myths by the use of variable and person-centred analyses. Our analysis reveals a clear association between higher pornography consumption, elevated Dark Tetrad traits, and increased acceptance of sexual aggression myths. This pattern suggests underlying mechanisms worth exploring further.

In a latent profile analysis, we identified three distinct groups: a majority with low scores on dark personality traits and low pornography use (49.5%), a second group with medium scores on all five measures (41.3%), and a smaller group with high scores on all traits, particularly everyday sadism (9.2%). Notably, higher Dark Tetrad trait scores were consistently associated with greater pornography consumption. These findings may reflect a combination of sensation-seeking tendencies and a possible alignment with desires for power and control found in certain pornographic content (Muris et al., 2020; Pineda et al., 2023; Trapnell et al., 2024).

Regarding the main objective of the study, the three groups showed significant differences in their acceptance of sexual aggression myths. Group 3, with higher Dark Tetrad scores and more frequent pornography consumption, demonstrated a notably higher acceptance of these myths. Our study corroborates previous research by Sánchez-Ruiz et al. (2021), indicating that the Dark Triad traits observed in our study are similarly associated with the acceptance of these myths, although in their case, they focused on rape myths. However, our study extends their work by highlighting that everyday sadism, which they did not assess, is similarly correlated with the acceptance of sexual aggression myths.

As for the analysis of the variables, we observed that Machiavellianism presents its highest correlations with the coercion, denial, and exoneration scales, indicating that those with high scores on this trait tend to use deception as a strategy for sexual purposes, and may use denial of the problem as a form of exculpation of their actions (Lyons et al., 2020). On the other hand, traits such as psychopathy and sadism also had their highest correlations with the denial and exoneration scales, which could be explained by the lack of empathy, deficits in cognitive responsiveness and lack of morality in people who score high on these scales, not understanding the severity of the problem for others and downplaying its importance (Moshagen et al., 2018; Turner et al., 2019; Willmott et al., 2024). These findings would align with the confluence model, emphasizing how these dark personality traits might shape individuals’ perceptions, which, in turn, might influence their tendencies to downplay or deny the severity of sexual aggression, perpetuating a cycle where these traits feed into and reinforce one another within the individual-environment dynamic (Seto et al., 2001; Turner et al., 2019).

In terms of predictive capacity, among the dark personality variables, Machiavellianism exhibited the strongest predictive capacity, whereas psychopathy and sadism showed minimal impact. Notably, consistent with a prior meta-analysis (Ferguson and Hartley, 2022), pornography consumption did not influence the acceptance of sexual aggression myths in the predictive model. Consequently, the disparities observed among profiles primarily align with differences in dark traits.

Trends in the variables analyzed also showed a relationship with the gender of the participants. As expected in relation to the findings of previous studies (Pineda et al., 2020, 2021a), males tended to present higher scores on the Dark Tetrad. Simultaneously, they consumed more pornography, which is also consistent with previous studies (Rostad et al., 2019; Borgogna et al., 2022), even though women were also represented in the three profiles. Finally, men also tended to accept more the myths of sexual aggression, especially those relating to denial of the problem, the use of coercive strategies, and the perception of over-support for victims. The latter could be because men do not identify with the possibility of being victims of sexual aggression and, therefore, tend to downplay the importance of the problem and its consequences. However, this relationship can also be explained by the wording of the items, which refers to the victimization of women (Gerger et al., 2007; Megías et al., 2011).

Previous literature reviews regarding sexual violence prevention have offered different suggestions about how to improve the programs designed to this end (e.g., by increasing the hours of the program, incorporating multiple sessions or focusing on bystanders; Kovalenko et al., 2020; Orchowski et al., 2020). However, there is limited literature regarding the disparities in the populations benefiting from these programs. In this regard, certain authors have emphasized the significance of targeting specific groups (DeGue et al., 2014). Based on our findings, we suggest enhancing program effectiveness by targeting individuals whose personality traits and patterns of pornography use indicate a higher susceptibility to accepting sexual aggression myths. This focused approach may effectively address and engage those more likely involved in such behaviours (Trottier et al., 2021). Our study’s findings may also have implications for the criminal justice system, particularly regarding how dark personalities and sexually aggressive attitudes influence juror decision-making. Research by Willmott (2018) and Lilley et al. (2023) shows that jurors with higher psychopathic traits and rape myth beliefs are more likely to render biased verdicts, even post-deliberation. Understanding these biases could lead to more effective jury selection and training reforms, potentially enhancing trial fairness and outcomes.

However, locating this group might be a challenge when it comes to non-research related fields, and thus non-anonymous situations (Galán et al., 2023). Therefore, several alternative measures are currently being developed to address the issues of self-report-based assessments in this field (Rico-Bordera et al., in press). Additionally, future research should focus on developing and validating tools that more accurately assess the impact of pornography consumption and examine how different types of pornography may influence attitudes toward sexual aggression. Furthermore, longitudinal studies could provide valuable insights into how these relationships evolve over time, offering a clearer understanding of causal pathways and the long-term effects of pornography consumption on sexually aggressive attitudes.

## 5 Limitations

The present research has several limitations. First, it’s important to note the limitation posed by the convenience sampling method employed. Specifically, this method resulted in a sample composition of 74% Western women, potentially impacting the broader generalizability of the findings. Another important limitation of the sample is the lack of data on participants’ sexual orientation, which may be an important factor influencing the variables of interest. Future research should consider including sexual orientation in the assessment to provide a more comprehensive understanding of its potential impact on the findings. Furthermore, as previously mentioned the study uses a cross-sectional design, which limits the ability to draw conclusions about causal relationships between Dark Tetrad traits, pornography consumption, and acceptance of sexual aggression myths. Longitudinal research could better clarify the directionality of these relationships over time.

As for the scales used, the use of self-reported instruments is a very common criticism of these studies given that participants may falsify the answers or offer responses that are marked by social desirability. This is even more significant in the case of socially reprehensible constructs. However, research in this field shows that people with high scores in the Dark Tetrad tend to be sincere, not caring about the image they show to the outside world since they have nothing to gain or lose in the field of research (Galán et al., 2023). Concerning the dark personality traits, it is relevant that they present important overlaps, reaching *r = 0*.70 between sadism and psychopathy, which may have contributed to the distinguished composition of the groups, without observing any intersections in the profiles. An additional limitation concerns the measurement of psychopathy and dark personality traits. The factorial structure and validity of these traits are debated, with scales like the PPTS and PPTS-R showing inconsistent results (Boduszek et al., 2018, 2022). This underscores the need for further research to improve and standardize psychopathy assessments. Regarding the instrument used to measure the acceptance of modern myths of sexual aggression, some scales, such as coercion or antagonism towards demands presented low internal consistency indices, which could have affected the results.

Finally, the last limitation concerns the measure used to assess pornography use, given the absence of validated measures for this purpose (Fisher and Kohut, 2020). The use of a more specific tool would be more appropriate given the typology of pornography that may influence the acceptance of aggression myths (i.e., degrading, violent, demeaning, objectifying, etc.). In this context, as mentioned in the discussion previous studies, have not found such a relationship between pornography use and sexual aggression, and even a weak correlation when speaking about violent pornography (Borgogna et al., 2022). However, it is worth mentioning that these small differences in the associations between the types of pornography and sexual aggression might be understood from a feminist perspective, considering a large part of pornography as violent or degrading towards women (De Miguel, 2021; Tranchese and Sugiura, 2021).

## 6 Conclusion

In conclusion, through an analysis of latent profiles, we have shown that there are different profiles according to the traits of the Dark Tetrad (i.e., narcissism, Machiavellianism, psychopathy, and sadism). Specifically, there is a profile (composed of 9.4% of our participants) with high scores on the Tetrad, a higher consumption of pornography, and which is more accepting of the myths of sexual aggression. This may serve as a possible starting point for sexual violence prevention programs, focusing efforts on those who are more likely to accept these myths.

## Data Availability

The datasets presented in this study can be found in online repositories. The names of the repository/repositories and accession number(s) can be found at: https://osf.io/8e3cs/?view_only=eb2b322ed19547a0945da8d36a1a8b8b, doi: 10.17605/OSF.IO/8E3CS.

## Footnotes

^1^ https://www.limesurvey.org
